# Degradation of different pectins by fungi: correlations and contrasts between the pectinolytic enzyme sets identified in genomes and the growth on pectins of different origin

**DOI:** 10.1186/1471-2164-13-321

**Published:** 2012-07-19

**Authors:** Isabelle Benoit, Pedro M Coutinho, Henk A Schols, Jan P Gerlach, Bernard Henrissat, Ronald P de Vries

**Affiliations:** 1 Microbiology & Kluyver Centre for Genomics of Industrial Fermentations, Utrecht University, Padualaan 8, Utrecht, 3584 CH, The Netherlands; 2 Architecture et Fonction des Macromolécules Biologiques, Aix-Marseille Université, CNRS UMR 7257, Case 932, 163 Av de Luminy, Marseille cedex 9, 13288, France; 3 Laboratory of Food Chemistry, Wageningen University, Bomenweg 2, Wageningen, 6703HD, The Netherlands; 4 Fungal Physiology, CBS-KNAW, Uppsalalaan 8, Utrecht, 3584 CT, The Netherlands

## Abstract

**Background:**

Pectins are diverse and very complex biomolecules and their structure depends on the plant species and tissue. It was previously shown that derivatives of pectic polymers and oligosaccharides from pectins have positive effects on human health. To obtain specific pectic oligosaccharides, highly defined enzymatic mixes are required. Filamentous fungi are specialized in plant cell wall degradation and some produce a broad range of pectinases. They may therefore shed light on the enzyme mixes needed for partial hydrolysis.

**Results:**

The growth profiles of 12 fungi on four pectins and four structural elements of pectins show that the presence/absence of pectinolytic genes in the fungal genome clearly correlates with their ability to degrade pectins. However, this correlation is less clear when we zoom in to the pectic structural elements.

**Conclusions:**

This study highlights the complexity of the mechanisms involved in fungal degradation of complex carbon sources such as pectins. Mining genomes and comparative genomics are promising first steps towards the production of specific pectinolytic fractions.

## Background

**Figure 1:**
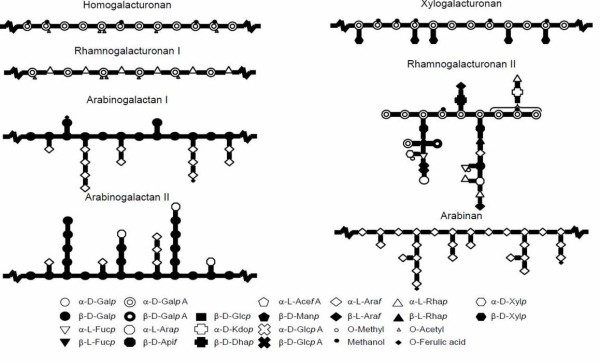
**Schematic representation of pectin structural elements [**[6]**]**.

Pectin is one of the major and one of the most complex plant cell wall components [1]. It forms a family of diverse polysaccharides composed of 17 different monomers containing more than 20 different glycosidic linkages [2,3]. Pectin is composed of six covalently linked substructures that are arranged to form a backbone substituted with side chains. Three pectic structural elements (homogalacturonan, rhamnogalacturonan-I, and substituted galacturonans) have been isolated from primary cell walls and were structurally characterized [4,5], (Figure 1, [6]). The architecture of pectin is dependent on the plant species and tissue, resulting in strong variations in the ratio of these different polysaccharides as well as their level of substitution [7,8]. The possible cross links between the different polysaccharide constituents are still not fully elucidated and several different models have been proposed to describe pectin architecture. In addition, some proteins are known to be associated to pectin, such as extensin in sugar beet pectin [9] and arabinogalactan proteins [10]. 

Pectin is an important and versatile additive in several food types because of its hydrocolloid character that makes it a gelling, stabilizing or thickening agent. The emulsification properties of sugar beet pectin due to a high content of acetyl groups and proteins make it as an interesting component for microencapsulation [11].

As a dietary fibre naturally present in plant products, it also provides nutritional benefits for the human diet. Derivatives of pectic polymers and oligosaccharides from pectin were shown to have positive effects on human health. These included immuno-regulatory effects in the intestine, lowering the blood cholesterol level, and slowing down the absorption of glucose in the serum of diabetic and obese patients [12,13]. A modified form of citrus pectin appears to be effective for a range of cancers at all stages of development and was patented as anti-cancer agent [14,15]. Moreover, pectin oligosaccharides proved to be good probiotic compounds [16][17].

Enzymatic hydrolysis of pectin has advantages over chemical hydrolysis as enzymes target specific linkages of the pectin molecules while chemical methods are less specific [18,19]. This is especially important when partial hydrolysis is required to produce specific fragments. To meet the demand for specific pectin-derived oligosaccharides, specialized mixtures of pectinolytic enzymes are required that cleave only those linkages necessary to produce these oligosaccharides, but do not further hydrolyse them.

Most of the current commercial pectinolytic enzyme mixtures are produced by filamentous fungi. These organisms are very efficient in the degradation of plant cell wall polysaccharides and use a broad set of enzymes to convert them into monomeric sugars that can be taken up as nutrients. However, the composition of these enzyme sets differs significantly between fungal species and this is also observed for the subset of pectinolytic enzymes. For instance *Rhizopus* spp. mainly degrades the homogalacturonan part of pectin, while Aspergilli produce enzymes to hydrolyse all pectic structural elements [20]. A better understanding of the correlation between the make-up of these enzyme sets and hydrolysis of different structural elements of pectin is likely to result in novel strategies to produce tailor-made pectinase preparations for the production of specific pectin-oligosaccharides.

The goal of this study was to compare the pectinolytic potential (based on their genome) of twelve fungi and their ability to use pectin (components) as a carbon source. These fungi were chosen because of their different life-styles and natural habitats. Some of these fungi have been studied in detail in relation to pectin degradation, while in others this topic is largely untouched. Four are ascomycete plant pathogens of which one is specific for cereals (*Magnaporthe oryzae*), while the other have a broad host plant range (*Giberella zeaeBotrytis fuckeliana, Sclerotinia sclerotiorum*) while the other eight are saprobes. The saprobic ascomycetes include model organisms (*Aspergillus nidulans, Trichoderma virens, Podospora anserina*) and industrial fungi that are commonly used for enzyme production (*Aspergillus nigerAspergillus oryzae*). The basidiomycete *Phanerochaete chrysosporium* has been the most intensively studied white rot fungus because of its powerful peroxidases and oxidases that are involved in lignin degradation [21], but little is known about its pectinolytic potential. The zygomycete *Rhizopus oryzae* is used in industrial fermentations for the production of lactic acid [22] and ethanol. It was found that the production rates of those two metabolites were affected by the activity of the pectinolytic enzymes [23,24]. The ascomycete *Aspergillus clavatus* is mainly studied in relation to the production of secondary metabolites [25,26] and the toxin patulin which may be associated with disease in humans and animals [27].

All fungi were grown on pectins from four different origins (Table 1) as well as on four structural elements of pectins: arabinan, galactan, rhamnogalacturonan I (RGI) and homogalacturonan (PGA). The ability to use the pectins and their structural elements as carbon source was compared to the pectinolytic potential of the fungi that was derived from their genome sequence.

**Table 1:** Sugar composition (mol %), acetylation and methylesterification degrees of the pectic polysaccharides used in this study

	**Fuc**	**Rha**	**Ara**	**Gal**	**Glc**	**Xyl**	**Uronic acids**	DA*	DM*	**reference**
**Soy pectin**	4	5	25	39	3	7	17	23	68	This study
**Sugar beet Beta**	-	5	7	11	0.4	1	73	14	30	This study
**Lemon pectin**	-	2	3	6	1	0.2	88	-	70	This study
**Apple pectin**	-	3	3	8	2	2	82	-	72	This study
**Galactan (potato)**	-	3	3	88	-	-	6	-	-	Megazyme
**Arabinan (sugar beet)**	-	2	88	3	-	-	7	-	-	Megazyme
**RGI (potato)**	-	20	3.3	12	-	1	62	-	-	Megazyme
**PGA (citrus)**	-	-	-	-	-	-	>97	-	-	Sigma

*Moles of acetyl (DA) groups or methyl (DM) esters groups per 100 moles of GalA.

## Results and discussion

### Genome annotation reveals significant differences in the pectinolytic potential of the twelve fungi

The Carbohydrate-active enzymes (CAZymes) related to pectin degradation of the twelve fungi tested in this study were identified using the CAZy annotation pipeline [28] and compared to each other (Table 2). Six of these fungi had a high number of CAZymes involved in pectin degradation while the other six had a lower content of these CAZymes. For instance *P. anserina* has 12 glycoside hydrolases, 7 polysaccharide lyases and 2 carbohydrate esterases involved in pectin degradation, while *A. oryzae* has 52 glycoside hydrolases (GH), 20 polysaccharide lyases (PL) and 9 carbohydrate esterases (CE) (Table 2). The CAZy annotations are based upon the protein models derived from the annotation of the genomes. As the pipelines from JGI and Broad are not identical in the identification of protein models, this may cause some differences in the numbers per family which may have had a small effect on the comparison. The CAZy families involved in pectin degradation and the activities that can be found within these families are listed in Table 3. Family PL11 (rhamnogalacturonan lyases) was not taken into account in the remainder of this study since only *A. nidulans* has one putative enzyme of this family which has not been biochemically characterised. 

**Table 2:** Pectinolytic glycoside hydrolases, polysaccharide lyases and carbohydrate esterases of the 12 fungal species used in this study

**CAZy family**	***S. sclerotiorum***	***B. fuckeliana***	***A. nidulans***	***A. clavatus***	***A. oryzae***	***A. niger***	***M. orysae***	***P. anserina***	***T. virens***	***G. zeae***	***P. chrysosporium***	***R. oryzae***
**GH2** LAC	1	1	3	0	1	0	3	3	1	3	0	0
**GH28** (Total)	16	18	10	2	20	21	3	0	6	6	3	18
- PGA	6	9	3	1	5	7	1	0	2	2	1	15
- PGX	2	2	3	1	2	5	1	0	2	3	1	3
- RHG	4	4	1	0	6	6	1	0	0	1	1	0
- RGX	3	2	2	0	5	2	0	0	2	0	0	0
- XGH	1	1	1	0	2	1	0	0	0	0	0	0
**GH35** LAC	4	4	4	3	7	5	0	1	1	3	3	1
**GH43** (Total)*	4	5	15	13	20	11	20	10	3	17	4	2
- ABF	0	0	3	0	3	0	6	2	0	0	0	0
- ABN	1	1	4	5	5	4	1	0	0	1	1	2
**GH78** RHA	4	8	9	0	8	8	3	1	3	7	0	0
**GH88**	0	1	3	0	3	1	1	0	3	1	1	0
**GH93** ABX	1	1	2	1	3	1	1	3	1	2	0	0
**GH105** URGH	1	1	4	3	2	2	3	0	1	3	0	0
**GH51** ABF	2	3	3	3	3	4	3	1	0	2	2	0
**GH53** GAL	2	2	1	0	1	1	1	1	0	1	1	0
**GH54** ABF	1	1	1	1	1	1	1	0	2	1	0	0
**GH127** ABF	0	1	1	1	0	0	0	0	0	1	0	0
**PL1** PEL, PLY	4	6	9	2	12	7	2	4	0	9	0	0
**PL3** PLY	0	2	5	1	3	0	1	2	0	7	0	0
**PL4** RGL	0	0	4	2	4	2	1	1	0	3	0	0
**PL9** PLY	0	0	1	0	1	0	0	0	0	1	0	0
**CE8** PME	5	5	3	7	5	3	1	1	2	6	0	6
**CE12** RGAE	3	3	2	1	4	2	3	1	0	3	0	0

* Total refers to all the GH43 including the activities not directly involved in pectin degradation such as β-xylosidase or exo-b-1,3-galactanase. Additional file 2.

**Table 3:** Activities of pectinolytic enzymes within CAZy families

**Glycoside hydrolases**	
**GH2**	β-galactosidase
**GH28**	
**PGA** **PGX** **RHG** **RGX** **XGH**	Endopolygalacturonase Exopolygalacturonase Endorhamnogalacturonase Exorhamnogalacturonase Xylogalacturonase
**GH35** **GH43**	β-galactosidase α-L-arabinofuranosidase, endo-α-1,5-L- arabinanase
**GH51**	*α*-L-arabinofuranosidase/endoglucanase
**GH53**	endo-1,4-β-galactanase
**GH54**	α-L-arabinofuranosidase/β-xylosidase
**GH78**	α-L-rhamnosidase
**GH88**	D-4,5 unsaturated β-glucuronyl hydrolase
**GH93**	exo-α-L-1,5-arabinanase
**GH105**	unsaturated rhamnogalacturonyl hydrolase
**GH127**	α-L-arabinofuranosidase
**Polysaccharide lyases**	
**PL1**	pectate lyase/exo-pectate lyase/pectin lyase
**PL3**	pectate lyase
**PL4**	rhamnogalacturonan lyase
**PL9**	pectate lyase/exopolygalacturonate lyase
**PL11**	rhamnogalacturonan lyase
**Carbohydrate esterases**	
**CE8**	pectin methylesterase
**CE12**	pectin acetylesterase/rhamnogalacturonan acetylesterase

### Distinct growth profiles of the twelve fungi on pectins and pectin structural elements can be in part explained by differences in CAZy content of their genome

**Figure 2:**
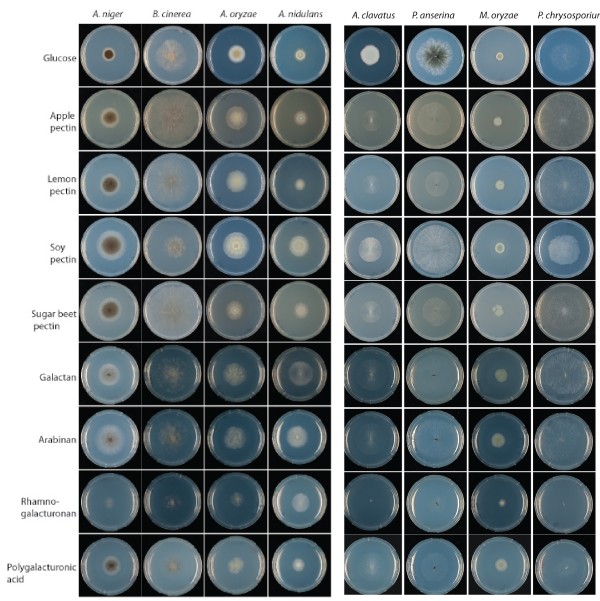
Correlation between growth profile and genome contents.

Growth on pectins from soy, apple, citrus and sugar beet and on four structural elements of pectin demonstrated different profiles for the twelve fungi of this study (Additional file 3). As the different species have different growth rates, glucose was used as standard for growth. Growth on the pectins and pectin structural elements was compared to growth on glucose and this relative growth difference was then used to compare the species to each other. *A. niger*, *A. oryzae*, *A. nidulans* and *B. cinerea* grew very well on pectin, while *A. clavatus*, *P. anserina* and *P. chrysosporium* grew poorly (Figure 2). The analysis of the CAZy families involved in pectin degradation in these species showed that the relative number of pectinases is lower for the species that do not grow well than for the species that grow well. *P. chysosporium*, *A. clavatus P. anserina* have 3, 2 and no GH28 members, respectively, while *A. oryzae*, *A. niger* and *A. nidulans* have respectively 20, 21 and 10 GH28 members with at least one gene encoding each activity of this family (Table 2). *A clavatus*, *P. chrysosporium* and *P. anserina* are also poorly equipped with families PL1, PL3, PL4 and PL9 when compared to the four other species.

These results show that the growth profiles on pectins and the overall pectinolytic enzyme content correlate. A similar correlation was observed in studies concerning xylan or cellulose degradation [29,30].

However, this correlation is less obvious when we compare growth on the structural elements of pectin to the presence of specific subsets of pectinases. For example, soy pectin does not contain homogalacturonan but a high proportion of rhamnogalacturonan I (Table 1). However, fungi that grow well on this pectin such as *A. nidulans, G. zeae* and *P anserina* do not always have an increased rhamnogalacturonan degrading potential (families GH28 RHG, RGX; GH51; GH54; GH93; GH43, ABN; PL1; PL3; CE8) compared to their homogalacturonan degrading potential (families GH28 PGA, PGX; PL4 and CE12). Interestingly, this also applies to *A. clavatus*, which, despite its overall poor growth, grows better on soy pectin than on the other substrates. And the fungi that have the higher rhamnogalacturonan degrading potential do not grow better on RGI. The best growth measured on RGI is for *T. virens* which has less rhamnogalacturonan degrading enzymes (only 10) than the other fungi of this study (18 in average). However, 10 enzymes are probably sufficient to degrade rhamnogalacturonan. More genes coding for the same putative activity could mean more possibilities/options to achieve an efficient degradation. So far, the regulation of the expression of the pectinolytic activities is not fully understood and is likely a determining factor for the production level of pectin related enzymes. For instance, most fungi that grow on cellulose produce a wide range of cellulases. Nonetheless, the best studied cellulolytic fungus, *Trichoderma reesei*, only produces a small number at very high levels [31].

**Figure 3:**
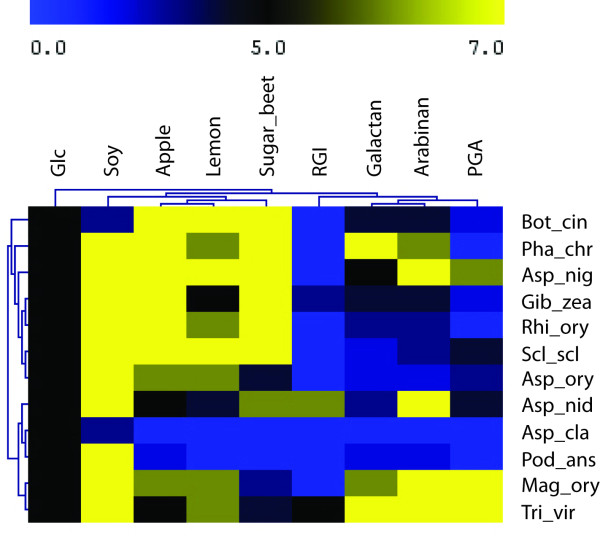
**Hierarchical clustering of the fungal species grown on pectins and pectic elements.** The growth of the 12 fungal species on the 4 pectins and 4 structural elements was used to generate a distance tree (see material and method for detail on measurements of the growth). The scores are represented by a colour scale from poor/no growth, deep blue to very good growth, yellow. The figure was edited using Mev [57].

In order to visualize better the growth profiles and compare them to each other, a double hierarchical clustering was calculated from the growth profile of the 12 fungi on the four pectins and four pectic structural elements (Figure 3). The fungal species clustered in three main groups. The largest group contains *B. cinerea*, *P. chrysosporium*, *A. niger*, *G. zeae*, *R. oryzae*, *S. sclerotiorum* and *A. oryzae*. The second group is made of *A. nidulans*, *A. clavatus* and *P. anserina* and the third group contains *M. oryzae* and *T. virens*. The substrates clustered in 2 main groups plus two separated branches. The first cluster contains polygalacturonic acid, arabinan and galactan, while the second cluster contains sugar beet, lemon and apple pectin. The two separated branches are soy pectin and potato rhamnogalacturonan I. The correlation between growth and genome content is clear at a two-dimension level, in that a high number of pectinases results in good growth rates. The complexity of the mechanisms involved appears when growth on pectin structural elements is compared to the presence of specific related activities.

In the paragraphs below, specific correlations will be discussed in more detail.

### *Rhizopus oryzae* is restricted to degradation of unbranched pectins

The CAZy annotation of *R. oryzae* identified 18 putative polygalacturonases (GH28), one putative β-galactosidase (GH35), 2 putative endo-arabinanases (GH43) and 6 putative pectin methyl-esterases (CE8). The *R. oryzae* genome contains a higher number of CAZymes related to pectin degradation than to the degradation of any other plant polysaccharide. Although it still has a rather small set of enzymes, this appears to be sufficient to (partially) degrade all four pectins to efficiently liberate carbon sources as good growth was observed on each of them. In a previous study, 3 of the 18 putative polygalacturonases were biochemically characterized as exo-polygalacturonases [24]. Both endo- and exo-polygalacturonases act on the homogalacturonan chains of the “smooth” region of pectin.

Interestingly, growth of *R. oryzae* was less good on the pectin structural elements. These results confirm the hypothesis that *R. oryzae* is restricted to the degradation of unbranched pectins. The analysis of Battaglia et al. [20] has shown that the enrichment of main-chain degrading enzymes of pectin in the *R. oryzae* genome was accompanied by a low number of accessory enzymes. This correlates with its life style as *R. oryzae* rot acts on PCW middle lamella of fruits and vegetables, which are rich in homogalacturonan (unbranched pectins).

On all substrates tested in this study, the growth profile of *R. oryzae* is similar to the growth profile of *S. sclerotiorum* that contains 16 GH28 with at least one gene of each subfamily (Figure 3). This indicates that not all subfamilies of GH28 are essential to degrade pectin and liberate sufficient amounts of monomers to enable growth.

*R. oryzae* and *S. sclerotiorum* cluster based on their growth profile with *G. zeae* and *A. oryzae* (Figure 3). These four fungi have 5, 6, 6 and 5 pectin methyl esterases (PME, CE8) respectively. Two other fungi have several CE8 members: *A. clavatus* (7) and *B. cinerea* (5). These enzymes are responsible for removing methyl-esters from pectin. Different sensitivities of fungal endopolygalacturonases to methyl esterification have been reported [32,33]. The methyl esterification degree of the four pectins of our study differs significantly, with 30% for sugar beet, 68% for soy, 70% for lemon and 72% for apple pectin. A high number of pectin methyl esterases could be an advantage to release galacturonic acid that can be directly metabolized in combination with polygalacturonases that act mainly on pectins with a low degree of methyl esterification. The phylogenetic tree of GH28 (Additional file 1) demonstrates that the 18 polygalacturonases from *R. oryzae* cluster together but separately from the polygalacturonases of the other fungi, including endo-polygalacturonases A, B, I and II from *A. niger* (PgaA, PgaB, PgaI, PgaII). PgaA and PgaB are more active on methylesterified pectin and are clustered together with PgaI and PgaII that prefer non-methylesterified pectin [32]. Although it can therefore not be concluded from the phylogenic tree, the high number of PMEs could indicate that *R. oryzae* polygalacturonases have higher affinity for non-methylated pectin.

### *Magnaporte oryzae* contains a small but complete set of pectinases

In contrast to the highly specialised enzyme set from *R. oryzae*, *M. oryzae* appears to possess nearly all required activities albeit at low numbers. It has at least one gene coding for 15 pectinolytic activities out of the 17 activities we analysed (Table 3). The two missing activities are exorhamnogalacturonase and xylogalacturonase. The CAZy families required to degrade galactan are β-galactosidase (GH35) and β-endogalactanase (GH53). *M. oryzae* has no characterized GH35 β-galactosidases and has only one putative β-endogalactanase, nevertheless grew equally well on galactan as on arabinan, lemon pectin and apple pectin. *M. oryzae* has also 8 GH2 members, but only three of those are candidate β-galactosidases and one is distantly related to β-galactosidases. These putative enzymes may explain the growth on galactan (Table 2). Alternatively the four α-arabinofuranosidases (GH51 and GH54) plus the three α-L-rhamnosidases (GH78) could be responsible for liberating the other sugars (arabinose, rhamnose) in galactan to support growth.

### The preference of *Trichoderma virens* for lemon pectin cannot be explained based on its genome content

In contrast to the other species, *T. virens* grew better on lemon pectin than on apple, soy and sugar beet pectin. It also showed good growth on galactan, arabinan and polygalacturonic acid. *T*. *virens* has 2 endopolygalacturonases, 2 exopolygalacturonases and 2 exorhamnogalacturonases and 3 β-glucuronyl hydrolases in its genome. These activities are required on substrates rich in uronic acids such as polygalacturonic acid (>97%), lemon (88%), apple (86%) and sugar beet (72.9%) pectins. The *T. virens* genome also contains 1 β-galactosidase, 3 α-rhamnosidases and 2 α-L-arabinofuranosidases that can be efficiently used to degrade galactan and soy pectin, containing 87% and 39% D-galactose, respectively, and potato RGI (20% L-rhamnose) and arabinan (88% L-arabinose).

Lemon and apple pectin have a very similar sugar composition. Lemon pectin contains a little less neutral sugars and a little more uronic acids than apple pectin (Table 1). Despite this similarity, G*. zeae, B. cinerea, A. nidulans, P. anserina* and *P. chysosporium* showed somewhat reduced growth on lemon pectin compared to apple pectin. No obvious difference in the CAZyme contents can explain this result. *T. virens* is the only fungus among the 12 fungi tested in this study that clearly show a preference for lemon pectin.

The poor growth of *T. virens* on arabinan is likely due to the absence of GH43 endoarabinanases, as several other enzymes involved in arabinan degradation were found in the *T. virens* genome. These are two α-arabinofuranosidases (GH54), and one exo-arabinanase (GH93). *R. oryzae* also has a small arabinan-degrading potential with no GH51, GH54 or GH93 members, and only 2 arabinan-related GH43 members. The growth profile of these two fungi on arabinan is less good than the growth profile of the other species that are richer in arabinan degrading enzymes such as *A. niger*, *A. nidulans* and *M. oryzae*.

### *Phanerochaete chrysosporium* may use PGA rather than PME to degrade high methyl esterified pectin

*P. chrysosporium* is poorly equipped with pectin degrading enzymes. Its genome contains only one candidate endopolygalacturonase, one exopolygalacturonase and one endorhamnogalacturonase in GH28, but does contain three GH35, two GH51, one GH53 and one GH88 members. Although it does not contain pectin methyl esterase it grew well on substrates that have a high methyl esterification degree such as soy pectin (DM 68) and similar but slightly less on apple (DM 72) and lemon pectin (DM 70). Two explanations are proposed. First, the endopolygalacturonase of *P. chrysosporium* may be more active on high methyl esterified pectin, which is supported by the clustering of this gene with *A. niger* PgaA and PgaB in the phylogenic tree (Additional file 3). PgaA and PgaB prefer pectins with a high methyl esterification degree [32]. Secondly, among the pectin structural elements, *P. chrysosporium* prefers galactan and arabinan. The endo-1,4-β-galactanase (GH53) and the β-galactosidase (GH35) release galactose from galactan, while the α-arabinofuranosidase (GH51) releases arabinose from arabinan. As soy pectin contains more galactose and arabinose than the other pectins, this may explain why growth on this pectin is somewhat better than on the other pectins.

### Exoarabinanase may be essential for rhamnogalacturonan I degradation to support fungal growth

Several pectinases can act on RGI. Exo- and endorhamnogalacturonases (GH28) and rhamnogalacturonan lyases (PL4) cleave the α-1-4-glycosidic bonds between L-rhamnose and D-galacturonic acids of the backbone. α-L-rhamnosidase (GH78) release the terminal non-reducing α-L-rhamnose residues of RGI, while α-L-arabinofuranosidases (GH51, GH54), endo- and exo-arabinanases (GH43, GH93), β-galactanases and β-galactosidases (GH53, GH35) act on the side chains.

**Figure 4:**
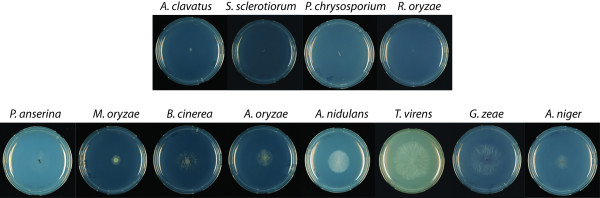
Growth on RGI related to GH93 presence.

Interestingly, the growth profiles demonstrate that the species able to grow on RGI all contain one or more genes encoding an exo-arabinanase (GH93) in their genome. This enzyme releases α-1,5-L-arabinobiose from the non-reducing end of RGI and is also active on linear L-arabinose oligomers. The reason for fungi to have more than one exo-arabinanase may lay in differences in the substrate specificity of these iso-enzymes. Having multiple slightly different enzymes for the same substrate will ensure efficient hydrolysis and a better utilization of the substrate. *P chrysosporium* and *R. oryzae* have no GH93. *A. clavatus* and *S. sclerotiorum* have one putative GH93 each but the sequences do not contain a signal sequence and the encoded enzymes are therefore likely not secreted. Those species cannot grow on RGI (Figure 4). Few studies have been reported on this enzymatic activity. The structure of the exo-arabinanase of *G. zeae* was recently described and a catalytic mechanism was proposed [34]. This activity is also described in two other fungal species, *Penicillium chrysogenum*[35], and *Chrysosporium lucknowense*[36], but they did not address its importance for RGI degradation. 

## Conclusions

The data from our study demonstrates that overall there is a clear correlation between the number of genes related to pectin degradation in fungal genomes and the ability of these fungi to grow on pectin. This correlation is less clear when the presence of specific pectin-acting enzymes is compared to growth on pectin structural elements. This indicates that different strategies can be employed by fungi to degrade these structural elements. While it is clear that a relative increase in pectin-related genes in general leads to better growth on pectin, the number of genes per enzyme activity does not necessarily correlate to improved growth on the part of pectin this activity is aimed at.

The differences in phenotype cannot be fully explained by genome content and regulation of the expression of the genes is certainly playing an important role. However, fungal regulatory systems of pectinolytic genes are far from fully elucidated. Galacturonic acid is considered to be the main inducer of pectinolytic genes, but other sugars (e.g. arabinose, rhamnose and galactose) are also involved in the induction of subsets of pectinolytic genes in *A. niger*[37]. So far only the arabinose-responsive regulator (AraR) has been identified [38]. This regulator is only present in the Aspergilli of this study, but not in the other species, which demonstrates that differences in pectin degradation between the species also exist at the regulatory level. Wubben et al. [39] postulated that *B. cinerea* endopolygalacturonases genes are regulated by four systems: basal expression, induction by pectic monomers, glucose repression and modulation by pH. Whether all four systems also affect pectin degradation in the other species is unclear at this point.

Alternatively, the currently available data on these enzymes is insufficient to understand the combined capacity to degrade different structural elements of pectin. One also has to keep in mind that approximately 40% of the genes of fungal genomes are not associated with a known function or precise substrate specificity [40]. In consequence it is possible that families not yet discovered intervene in the breakdown of specific pectin structural elements. Two recent examples are (i) the identification and characterization of the α-glucuronidase from *S. commune* that was assigned to a novel glycoside hydrolase family (GH115) [41] and (ii) the GH127 family created after Fujita et al. [42] who characterized a novel β-L-arabinofuranosidase from *Bifidobacterium longum*. In addition, activities currently suggested for members of established CAZy families may be incorrect due to lack of biochemical support, changing the overall comparison of the fungal species.

From the four pectins tested, soy and sugar beet pectin are most different in their composition. Proteomics on the secretome of the 12 fungi grown on these pectins would give an overview of which enzymes are responsible for breaking down these different pectin structures. Future studies could also include other pectins with different properties, such as hop pectin which has a lower degree of methyl esterification than apple and citrus pectin and a relatively high degree of acetylation and neutral sugar content.

 This study demonstrates the large possibilities that fungal species offer to efficiently degrade different types of pectin. Pectic structures are extremely diverse, depending on the origin of the plant and on the plant tissue. Fungi evolved in specific biotopes and developed tools adapted to colonize broad range of substrates such as pectins. Interesting points were also observed at the structural element level. In particular, strong indications were found for an important role of exo-arabinanase in RGI degradation.

 Our data is a first step to being able to select fungi, which produce highly specific pectinase mixtures. These could be aimed at the production of desired polymers and oligosaccharides, such as those with a positive effect on human health or better colloid properties. In addition, the differences observed between the species can also be used to identify pectinolytic genes that are missing or insufficiently expressed in the current industrial pectinases producers.

## Methods

### Chemicals and media

Aspergillus minimal medium was described by de Vries et al. [43]. Culture conditions and minimum media for *P. anserina, S. sclerotinia* and *T. virens* were used as respectively described by Silar [44] Cruickshank [45] and Deane et al. [46]. 1% of the carbon sources (except glucose, see below) were added to the minimal medium containing 1.5% agarose (Merck,101614) For glucose, 25 mM was used as final concentration. The pH of the medium was adjusted to 6.0 and autoclaved.

Soy pectin (soybean soluble polysaccharide (SSPS; Soyafibe-S-DA100) was obtained from Fuji Oil Co. Ltd (Osaka, Japan) and prepared from the residue of soy protein extraction. Apple and lemon pectin were kindly provided by Degussa Texturant Systems (Baupte, France). Sugar beet pectin was obtained from Copenhagen pectin A/S (Lille Skensved, Denmark). Potato Galactan, sugar beet Arabinan, potato Rhamnogalacturonan I were obtained from Megazyme (Bray, Ireland)., Citrus polygalacturonic acid and glucose were obtained from Sigma–Aldrich.

### Strains and growth conditions

The fungal strains and culture conditions are listed in Table 4. For *A. niger* the commonly used lab strain N402 [47] was used and compared to the genome of the strain sequenced by JGI which is highly similar. The centre of the plates was inoculated with 2 μl of a suspension of 500 spores/μl or with a small agar plug containing mycelium (1 mm diameter) transferred from the edge of a vigorously growing 3-days-old colony. The cultures were incubated at 25°C or 30°C as indicated in Table 4. Mycelium density and colony diameter were measured daily. Colony morphology pictures were taken after 3 or 5 days depending on the growth rate of the fungi. The growth test was performed in triplicate for each strain. 

**Table 4:** Culture conditions

**Species**	**Strain number**	**Reference for genome**	**Medium**	**Inoculation**	**Growth T (°C)**
*Sclerotinia sclerotiorum*	ATCC18683	[60]	MM Sclerotinia	Mycelial plug	25
*Botryotinia fuckeliana*	B05.10	[60]	MM Aspergillus	10^3^ sp	25
*Aspergillus nidulans*	FGSC A4	[61]	MM Aspergillus	10^3^ sp	30
*Aspergillus clavatus*	NRRL1	http://www.broadinstitute.org	MM Aspergillus	10^3^ sp	30
*Aspergillus oryzae*	RIB40	[62]	MM Aspergillus	10^3^ sp	30
*Aspergillus niger*	N402	[63]	MM Aspergillus	10^3^ sp	30
*Magnaporthe oryzae*	70-15	[64]	MM Aspergillus	10^3^ sp	30
*Podospora anserina*	S mat+	[65]	MM Podospora	Mycelial plug	30
*Trichoderma virens*	Gv29-8	genome.jgi-psf.org	MM Trichoderma	10^3^ sp	30
*Gibberella zeae*	FG05491.1	[66]	MM Aspergillus	Mycelial plug	30
*Phanerochaete chrysosporium*	RP78	[67]	MM Aspergillus	Mycelial plug	25
*Rhizopus oryzae*	99-880	[68]	MM Aspergillus	10^3^ sp	25

### Growth evaluation

Growth was independently scored by two persons. The mycelium density was evaluated by visual inspection of the colonies and combined with the diameter to obtain a score between 1 and 10. Glucose was taken as the internal reference and scored as 5. A better growth compared to the reference was scored between 6 and 10 when a poor growth was scored between 0 (no growth) and 4. Sporulation was taken in account as a positive criterion of growth.

### Sugar composition determination

Sugar composition was determined after Saeman Hydrolysis. After a prehydrolysis step using 72% w/w sulphuric acid at 30°C for 1 h the samples were hydrolysed with 1 M sulphuric acid at 100°C for 3 h. Afterwards the sugars were derivatised as alditol acetates and determined by gas chromatography [48] using inositol as internal standard.

#### Uronic acid content

The total uronic acid content was determined with the automated m-hydroxydiphenyl assay [49].

#### Degree of acetylation and methyl esterification

Degree of acetylation and methyl esterification were determined by HPLC after hydrolysis with 0.4 N sodium hydroxide in isopropanol/water (50/50 v/v) [50]. The degree of acetylation and methyl esterification are calculated as mole methyl/acetyl groups per 100 mole galacturonic acid. One mole galacturonic acid can carry only one mol methyl esters and two moles acetyl ester.

### CAZy annotation

The identification step of CAZymes followed the procedures previously described [29] where sequences are subject to BlastP analysis [51] against a library composed of modules derived from the CAZy database. The positive hits are then subjected to a modular annotation procedure that maps the individual modules against libraries of catalytic and carbohydrate models derived from CAZy using BlastP or Hidden Markov models [52,53]. The results are augmented with signal peptide, transmembrane, and GPI predictions by human curators [54-56]. The fragmentary models and all models suspected of splicing prediction errors are identified. The functional annotation step involves BlastP comparisons against a library modules derived from biochemically characterized enzymes [29].

### Hierarchical clustering of pectin degradation capacity and phylogenetic analysis

To visualize the growth profile of the 12 fungal species on the 4 pectins and 4 structural elements, a Pearson correlation matrix was generated by using MeV, Multiexperiment Viewer [57].

For the construction of the phylogenetic trees of the GH families the sequences of the family members were aligned using Muscle (version 3.8.31) [58] after which the alignment was used to generate a bootstrapped minimal evolution tree (1000 bootstraps) using MEGA4 [59].

## Competing interests

The authors declare that they have no competing interests.

## Authors’ contributions

IB performed the experiments, analyzed the data and drafted the manuscript. PMC and BH performed comparative genomic analysis. HAS supplied part of the experimental material. RPdV and IB designed the study. All authors contributed to the interpretation of the data. All authors read and approved the final version of the paper.

## Supplementary Material

Additional file 1 Phylogeny of the GH28 family. Click here for file

Additional file 2 Phylogeny of the GH43 family. Click here for file

Additional file 3 Growth profiles of the 12 fungi on the 4 pectins, the 4 structural elements and glucose. Click here for file
