# Supplementary material for: Degradation of different pectins by fungi: correlations and contrasts between the pectinolytic enzyme sets identified in genomes and the growth on pectins of different origin
Source: BMC Genomics. 2012 Jul 19;13:321. doi: 10.1186/1471-2164-13-321 (PMC3460790; doi:10.1186/1471-2164-13-321)

Additional file 2. Phylogeny of the GH43 family from the 12 fungal species used in this study.
Bootstrap values are indicated at the branch points. Amino acids sequences were aligned with CLUSTAL X software, version 2.0.
Phylogenetic tree was constructed by the neighbor-joining method (Saitou and Nei, 1987), using MEGA software, version 4.0 (Tamura et al., 2007).
Bootstrap values were calculated based on 100 replicates of the data. All sequences were obtained from CAZy pipeline.
The following activities are mentioned: α-L-arabinofuranosidase (ABF), arabinanase (ABN), β-xylosidase (BXL).

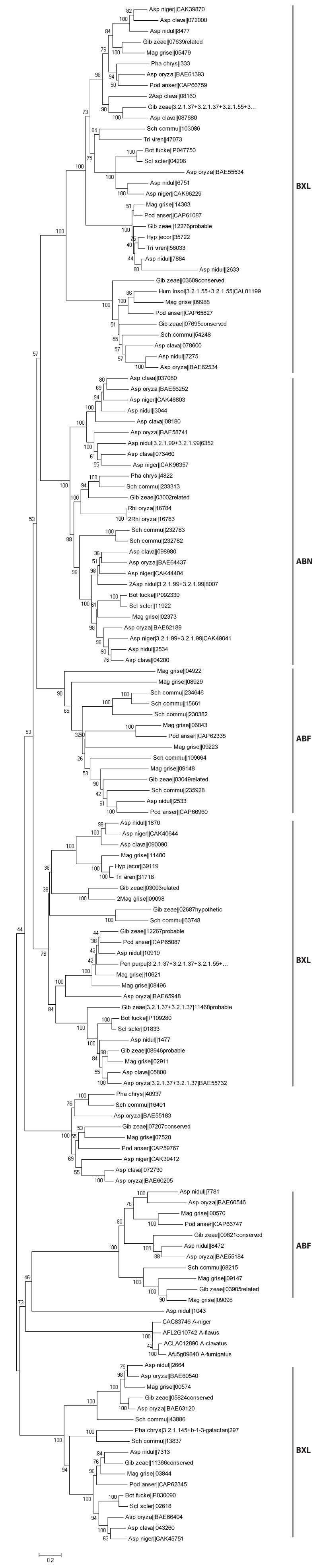

Supplement: Additional file 2 — Phylogeny of the GH43 family. [file 1471-2164-13-321-S2.pdf]
