# Supplementary figures and images for: Degradation of different pectins by fungi: correlations and contrasts between the pectinolytic enzyme sets identified in genomes and the growth on pectins of different origin
Source: BMC Genomics. 2012 Jul 19;13:321. doi: 10.1186/1471-2164-13-321 (PMC3460790; doi:10.1186/1471-2164-13-321)

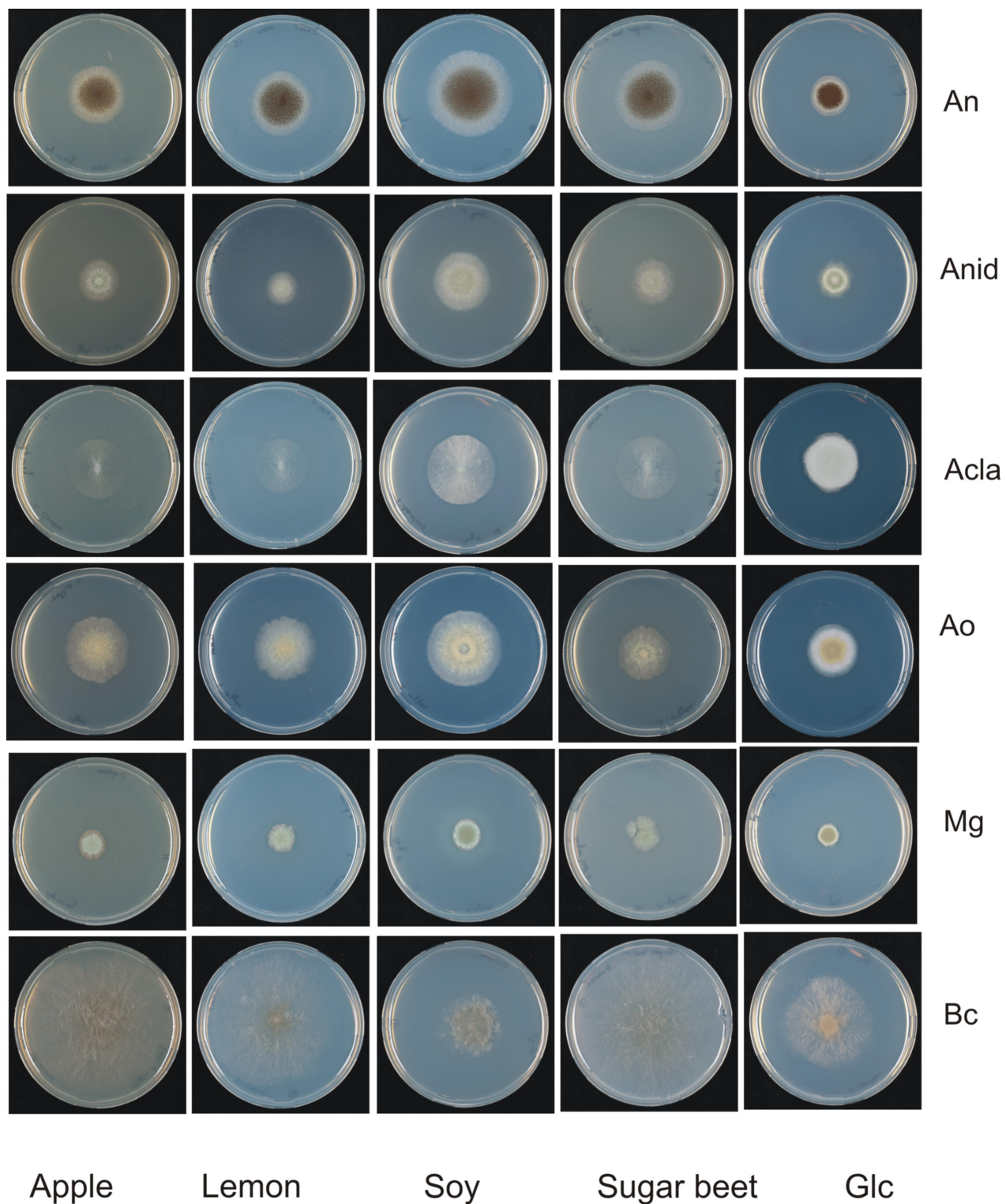

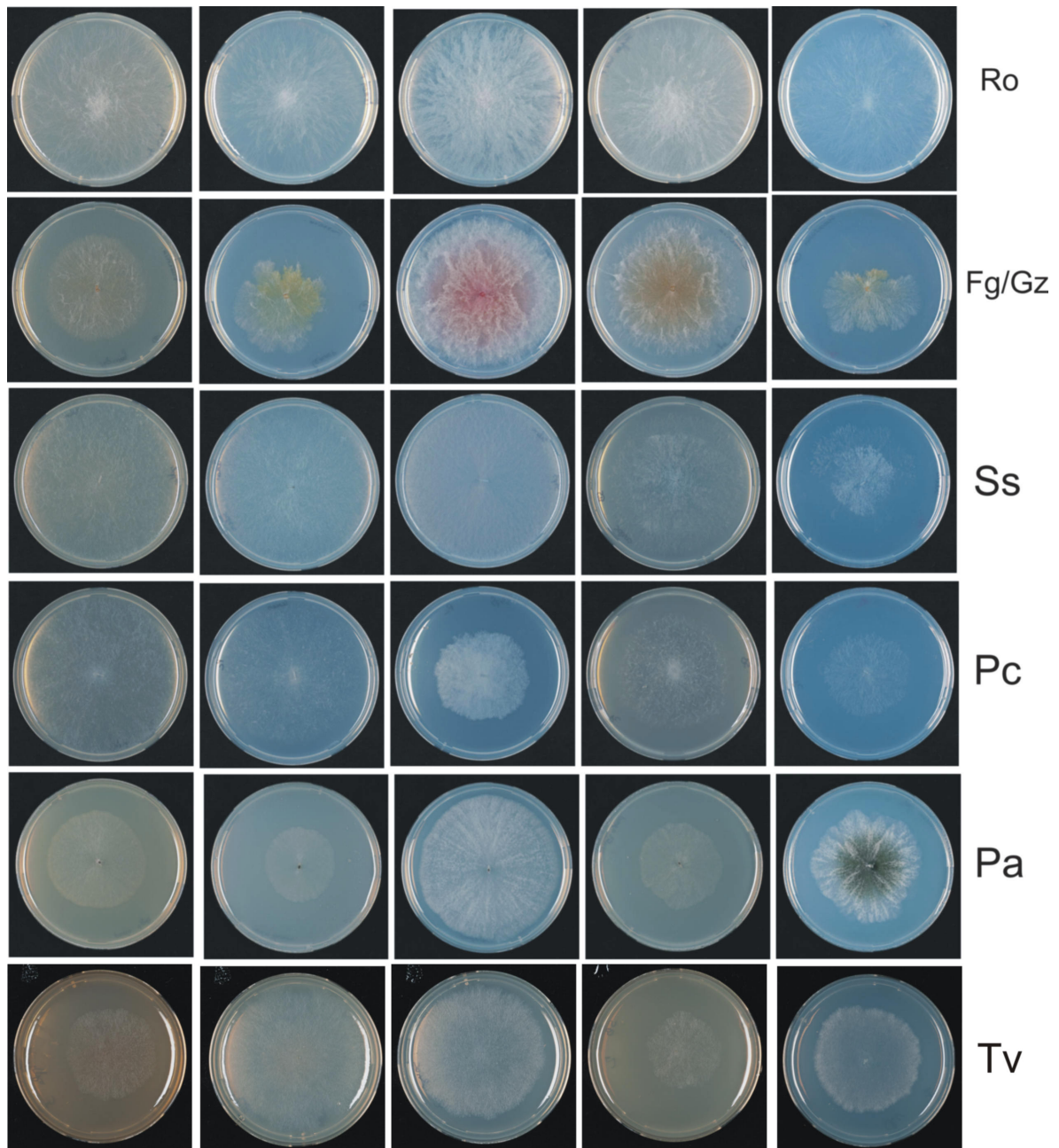

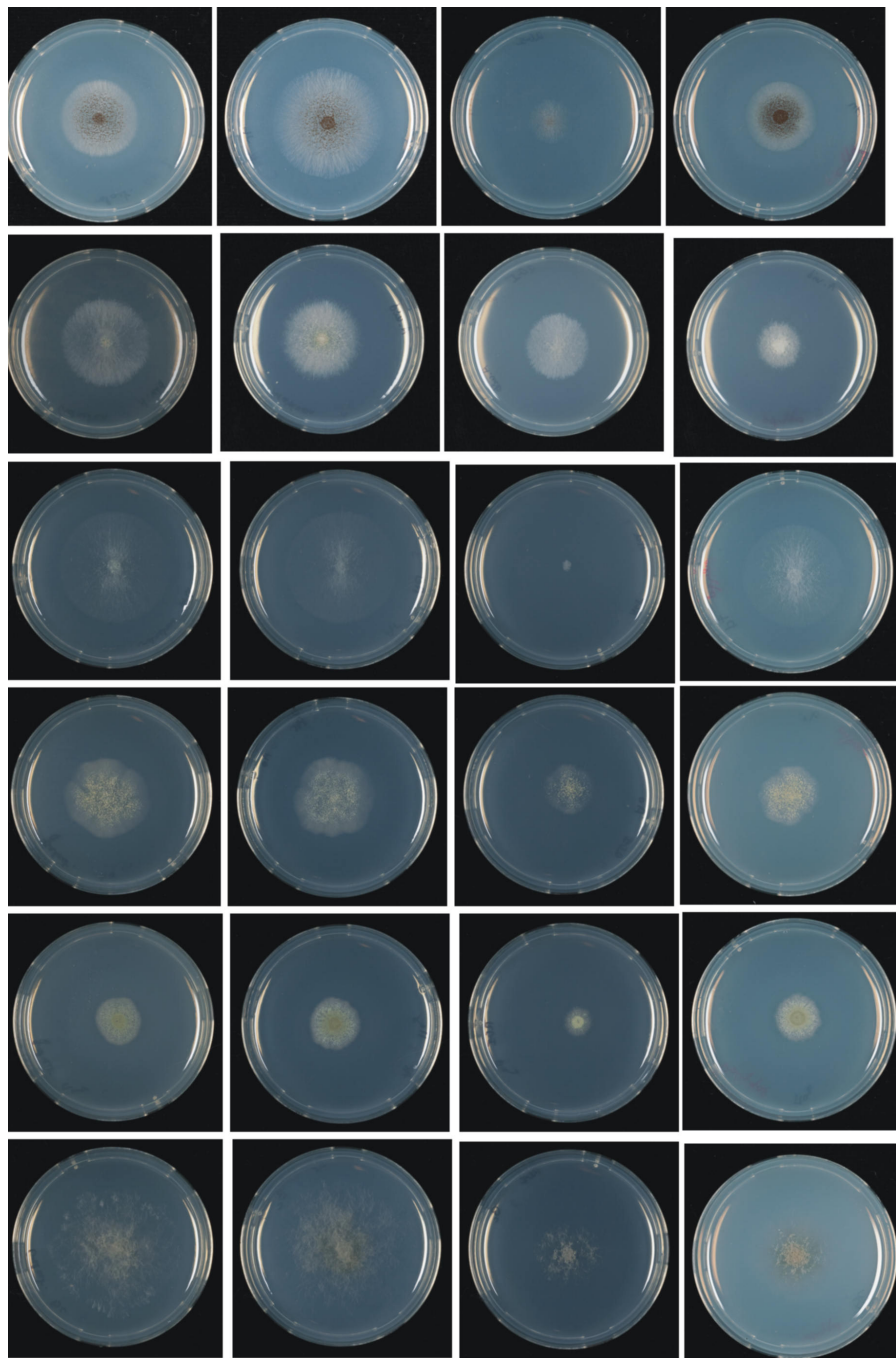

An

Anid

Acla

Ao

Mg

Bc

Galactan

Arabinan

RGI

PolyGal A

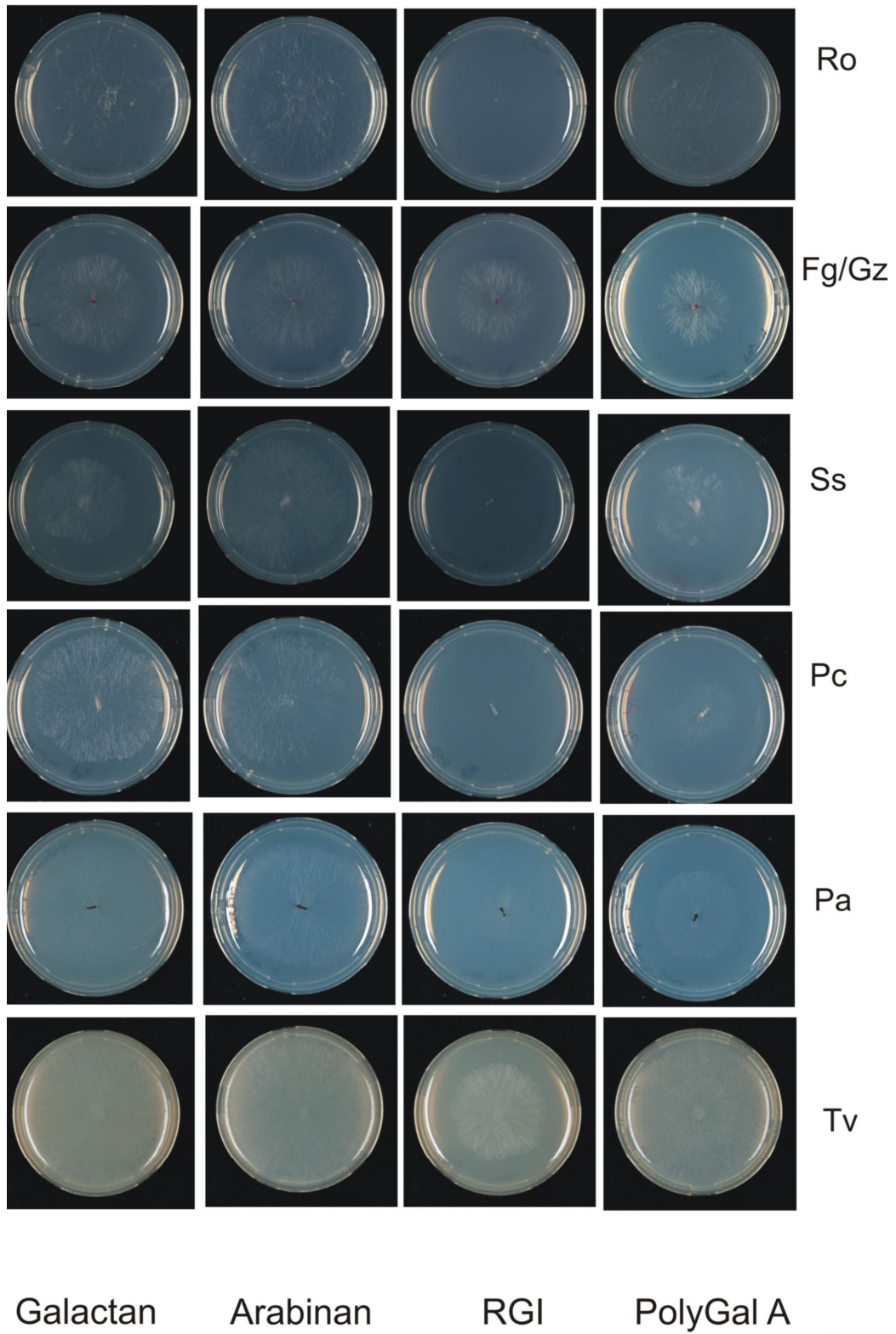

Supplement: Additional file 3 — Growth profiles of the 12 fungi on the 4 pectins, the 4 structural elements and glucose. [file 1471-2164-13-321-S3.pdf]
